# A Prospective Study of Etiology of Childhood Acute Bacterial Meningitis, Turkey

**DOI:** 10.3201/eid1407.070938

**Published:** 2008-07

**Authors:** Mehmet Ceyhan, Inci Yildirim, Paul Balmer, Ray Borrow, Bunyamin Dikici, Mehmet Turgut, Nese Kurt, Aysel Aydogan, Cigdem Ecevit, Yasar Anlar, Ozlem Gulumser, Gonul Tanir, Nuran Salman, Nezahat Gurler, Nevin Hatipoglu, Mustafa Hacimustafaoglu, Solmaz Celebi, Yavuz Coskun, Emre Alhan, Umit Celik, Yildiz Camcioglu, Gulten Secmeer, Deniz Gur, Steve Gray

**Affiliations:** * Hacettepe University, Ankara, Turkey; † Health Protection Agency North West, Manchester, United Kingdom; ‡ Dicle University, Diyarbakir, Turkey; § Firat University, Elazig, Turkey; ¶ Dr. Behcet Uz Children’s Hospital, Izmir, Turkey; # Mayis University, Samsun, Turkey; **Sami Ulus Children’s Hospital, Ankara; †† Istanbul University, Istanbul, Turkey; ‡‡ Uludag University, Bursa, Turkey; §§ Gaziantep University, Gaziantep, Turkey; ¶¶ Cukurova University, Adana, Turkey; ## Pediatric Microbiology Laboratory, Ankara

**Keywords:** Bacterial meningitis, epidemiology, *Neisseria meningitidis*, serogroup W-135, meningococcal vaccine, research

## Abstract

Determination of the etiology of bacterial meningitis and estimating cost of disease are important in guiding vaccination policies. To determine the incidence and etiology of meningitis in Turkey, cerebrospinal fluid (CSF) samples were obtained prospectively from children (1 month–17 years of age) with a clinical diagnosis of acute bacterial meningitis. Multiplex PCR was used to detect DNA evidence of *Streptococcus pneumoniae*, *Haemophilus influenzae* type b (Hib), and *Neisseria meningitidis*. In total, 408 CSF samples were collected, and bacterial etiology was determined in 243 cases; *N. meningitidis* was detected in 56.5%, *S. pneumoniae* in 22.5%, and Hib in 20.5% of the PCR-positive samples. Among *N. meningitidis*–positive CSF samples, 42.7%, 31.1%, 2.2%, and 0.7% belonged to serogroups W-135, B, Y, and A, respectively. This study highlights the emergence of serogroup W-135 disease in Turkey and concludes that vaccines to prevent meningococcal disease in this region must provide reliable protection against this serogroup.

## Abstract 2

Vaccines to prevent bacterial meningitis in this region must provide reliable protection against serogroup W-135.

Acute bacterial meningitis is one of the most severe infectious diseases, causing neurologic sequelae and accounting for an estimated 171,000 deaths worldwide per year (*1*,*2*). Although most disease occurs in infants, the societal impact is also important because of the continued high incidence in healthy older children and adolescents. Despite many new antibacterial agents, bacterial meningitis fatality rates remain high, with reported rates between 2% and 30% (*3*,*4*). Furthermore, permanent sequelae, such as epilepsy, mental retardation, or sensorineural deafness are observed in 10%–20% of those who survive (*5*,*6*).

The 3 most common etiologic agents are *Haemophilus influenzae* type b (Hib), *Streptococcus pneumoniae*, and *Neisseria meningitidis*, which account for 90% of reported cases of acute bacterial meningitis in infants and children >4 weeks of age (*7*,*8*). Hib meningitis is a disease affecting primarily young children; most of the cases occur in children 1 month to 3 years of age (*3*,*8*). The use of Hib conjugate vaccines has reduced the incidence of, or even virtually eliminated, invasive Hib disease in some industrialized countries (*7*,*8*). *S. pneumoniae* is a major cause of childhood bacterial meningitis in countries where Hib disease has been eliminated by vaccination (*9*). It is the second most frequently reported cause of septic meningitis in some European and sub-Saharan African countries, after meningococcal cases (*4*,*9*).

*N. meningitidis* is now considered to be the leading cause of bacterial meningitis in many regions of the world, causing an estimated 1.2 million cases of bacterial meningitis and sepsis worldwide each year (*10*,*11*). Meningococci are classified into 13 serogroups based on the antigenic properties of their capsular polysaccharide; however, nearly all disease is caused by 5 serogroups: A, B, C, W-135, and Y. The epidemiology of *N. meningitidis* varies by serogroup; currently, serogroups A, B, and C account for >90% of meningococcal disease worldwide (*12*). However, the epidemiologic landscape is constantly changing, and with increasing international travel and cross-border migration, the epidemiology of this disease will remain dynamic. Currently, serogroups A and C predominate throughout Asia and Africa, whereas serogroups B and C are responsible for most cases in Europe and North America (*11*,*13*–*18*). In several countries, including the United States, the proportion of disease caused by serogroup Y has increased over the past decade, where it now accounts for approximately one third of meningococcal cases (*19*). Serogroup W-135 has also recently emerged in some parts of the world, primarily in the Middle East and Africa, in some instances causing large epidemics (*20*).

The annual Hajj pilgrimage to Mecca is a major international event; ≈2 million people from around the world gather in one place, where the extreme crowding provides an ideal environment for transmission of meningococcal carriage. On several occasions, meningococcal disease outbreaks have subsequently spread worldwide by returning pilgrims. A major serogroup A meningococcal disease epidemic occurred in the 1980s, affecting Muslim pilgrims initially, followed by populations in other Middle Eastern and African countries (*21*). After this epidemic, Hajj pilgrims were vaccinated with a bivalent (A and C) meningococcal polysaccharide vaccine before entering Saudi Arabia. With the emergence of serogroup W-135 meningococcal disease among Hajj pilgrims in the Middle East during 2000 and 2001 (*20*), vaccine recommendations for pilgrims were changed to quadrivalent (A, C, W-135, and Y) meningococcal polysaccharide vaccine in 2002 (*22*).

Global surveillance of confirmed meningococcal cases, including surveillance of the diversity of causative strains, is essential to managing disease and developing vaccines. This study was undertaken to determine the current etiology of bacterial meningitis in Turkey, with particular emphasis on serogroup distribution of meningococci. Turkey is a predominantly Muslim country, and as such epidemics originating at the Hajj may have an effect on the national epidemiology. Although limited epidemiologic studies are available, cases of invasive meningococcal disease as well as carriage of serogroup W-135 have been reported in Turkey (*23*–*25*). This finding is in contrast to Western Europe, where the incidence of W-135 disease remains low. Turkey has no surveillance system for bacterial meningitis, and exact rates of meningococcal disease and serogroup distribution are unknown. Reliable surveillance data from countries such as Turkey are vital to understand, and better anticipate, the constantly changing landscape of bacterial meningitis and meningococcal disease.

## Materials and Methods

### Study Design

**Figure 1:**
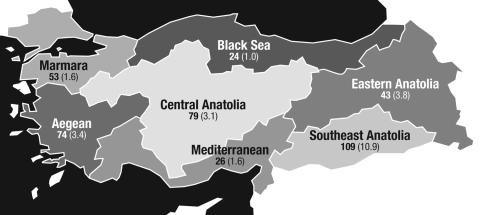
Meningitis cases by geographic region of Turkey. The number of suspected meningitis cases included in the study per region is shown in **boldface**, with the region-specific estimated incidence rate of laboratory-confirmed meningitis (per 100,000 population) shown in parentheses. In total, 408 children were diagnosed with suspected acute bacterial meningitis. Bacterial meningitis was confirmed by PCR, culture, or latex agglutination test in 243 cases. Region-specific incidence rates ranged from 1.0 to 10.9/100,000 population.

From February 16, 2005, through February 15, 2006, active surveillance of acute bacterial meningitis among children admitted to 12 participating hospitals was undertaken. Turkey is divided into 7 geographic areas (Figure 1). Twelve health centers in 9 cities located in all of these 7 geographic regions were selected to represent the population characteristics of the country. Two centers from each of the 3 biggest cities and 1 center from each of the other cities were included. Each health center served as a referral center for its region in the field of pediatric diseases. The centers serve ≈32% of the entire pediatric population of Turkey. Approval was obtained from the ethical committees of the participating centers and Ministry of Health.

In each hospital, suspected cases of acute bacterial meningitis were identified by a pediatrician, based on the following criteria: any sign of meningitis (fever [axillary measurement >38°C], vomiting [>3 episodes in 24 h], headache, meningeal irritation signs [bulging fontanel, Kernig or Brudzinski signs, or neck stiffness]) in children >1 year of age; fever without any documented source; impaired consciousness (Blantyre Coma Scale <4 if <9 months of age and <5 if >9 months of age) (*26*); prostration (inability to sit unassisted if >9 months of age or breastfeed if <9 months of age) in those <1 year of age; and seizures (other than those regarded as simple febrile seizures with full recovery within 1 h). For each suspected case, demographic data, predominant clinical signs and symptoms, prior history of use of antimicrobial agent, and laboratory results were recorded by using a standardized case report form.

Cerebrospinal fluid (CSF) samples were obtained from all patients <17 years of age with clinical suspected meningitis. Patient samples were included in further analyses if the CSF had 1) >10 leukocytes/mm^3^ in the CSF, and/or 2) higher CSF protein levels than normal for the patient’s age, and/or 3) lower CSF glucose levels than normal for the patient’s age. In addition to these patients, all who had a positive CSF culture, PCR, Gram stain, or antigen detection test result were also included in the study. No neonates (<1 month of age) were included in the study since the pathogens of neonatal meningitis were expected to be different than those of the older case-patients (*3*).

### CSF Cultures and Bacterial Isolates

CSF cultures, Gram stain, and latex agglutination tests (Wellcogen Bacterial Antigen Kit, Lenexa, KS, USA) were performed in the local hospitals. A CSF sample (minimum 0.5 mL) from each patient was kept at –20°C until transportation to the Central Laboratory (Hacettepe University, Ankara, Turkey) for PCR analysis. CSF samples and, if available, bacterial isolates were sent to the Central Laboratory, where all isolates were recultured on chocolate and blood agars and grown at 37°C in 5% CO_2_. Suspected meningococcal colonies were characterized by Gram stain, oxidase test, and rapid carbohydrate utilization test (Gallerie Pasteur, Pasteur Merieux, Lyon, France). The microbiology laboratory records were cross-checked in each hospital for missing data. The phenotypic determination, based on the antigenic formula (serogroup:serotype:serosubtype) of meningococcal isolates, was performed by standard methods in the Meningococcal Reference Unit, Health Protection Agency, Manchester, United Kingdom (*18*,*20*–*25*,*27*).

### DNA Isolation

All samples collected in the Central Laboratory were kept at –80°C and were thawed immediately before each test. Bacterial colonies were suspended in 500 μL sterile double-distilled water and vortexed. Bacterial suspensions and CSF were boiled for 3 min at 100°C, then centrifuged for 5 min at 10,000 × *g*, and the supernatant was retained. DNA concentration was estimated spectrophotometrically, and 15 μL (≈50 ng) was used in each final reaction mixture.

### PCR Amplification

For the simultaneous identification of bacterial agents, single tube, multiplex PCR assay was performed. The specific gene targets were *ctrA*, *bex,* and *ply* for *N. meningitidis,* Hib, and *S. pneumoniae,* respectively (*28*). In each assay, the final reaction mixture of 50 μL contained 15 μL (≈50 ng) DNA, 1× PCR buffer, 3 mmol/L MgCl_2_, 200 μmol/L of each dNTP (AB Gene, Epsom, UK), 0.6 μmol/L of each corresponding oligonucleotide primer (Sigma Aldrich, Seezle, Germany) as described (*28*) and 1 U of Taq polymerase (AB Gene). The PCR was performed by using a DNA thermal cycler (Perkin-Elmer Cetus, Emeryville, CA, USA model 9600) under the following conditions: a first cycle of denaturation at 95°C for 5 min followed by 35 cycles of 95°C for 25 s, 57°C for 40 s, and 72°C for 60 s.

Among the samples positive for *N. meningitidis,* serogroup prediction (A, B, C, W-135, and Y) was based on the oligonucleotides in the *siaD* gene for serogroups B, C, W-135, and Y and in orf-2 of a gene cassette required for serogroup A (*28*). For serogroup determination, amplification reactions (50 μL) contained 15 μL of DNA, 60 mmol/L Tris-HCl (pH 8.8), 17 mmol/L (NH4)_2_SO_4_, 5 mmol/L MgCl_2_, 0.5 mmol/L of each dNTP, 0.3 μmol/L corresponding oligonucleotides, and 1 U of Taq polymerase. The PCR conditions were as follows: denaturation at 94°C for 3 min, followed by 35 cycles of 92°C for 40 s, 55°C for 30 s, and 72°C for 20 s in a DNA thermal cycler. A final extension cycle at 72°C for 10 min was then performed (*29*).

All amplicons were analyzed by electrophoresis on standard 3% agarose gels and visualized by using UV fluorescence. A negative control consisting of distilled water and a positive control consisting of a reference strain (*S. pneumoniae* ATCC 49613, Hib ATCC 10211, *N. meningitidis* serogroup C L94 5016 also known as C11 [C:16:P1.7–1,1], serogroup A M99 243594 [A:4,21:P1.20,9], serogroup Y M05 240122 [Y:NT:P1.5], serogroup W135 M05 240125 [W135:2a:NST], serogroup B M05 240120 [B:NT:NST]) were analyzed simultaneously.

### Statistical Analysis

Continuous variables were compared by the Student *t* test and categorical variables with χ^2^ or Fisher exact tests. A 2-tailed p value <0.05 was considered significant. All statistical analysis was performed with SPSS version 11.5 (SPSS Inc, Chicago, IL, USA).

## Results

### Meningitis Cases

In total, 408 children were hospitalized with a clinical diagnosis of meningitis during the study period (Figure 1), and a CSF sample from each patient was obtained. The distribution of these suspected cases according to the geographic regions was as follows: 109 (26.7%) in Southern Anatolia, 74 (18.1%) in Aegean region, 79 (19.6%) in Central Anatolia, 53 (13.0%) in Marmara region, 43 (8.7%) in Eastern Anatolia, 24 (5.9%) in Black Sea region, and 26 (6.4%) in Mediterranean region. The mean age of the 408 children was 4.8 years (standard deviation 4.1 years), and the boy-to-girl ratio was 1.5:1. Of 408 patients diagnosed with acute bacterial meningitis, 20 (4.9%) died and 14 (5.7%) of these deaths were among patients with laboratory-confirmed cases.

### Laboratory-Confirmed Meningitis Cases and Etiology

**Figure 2:**
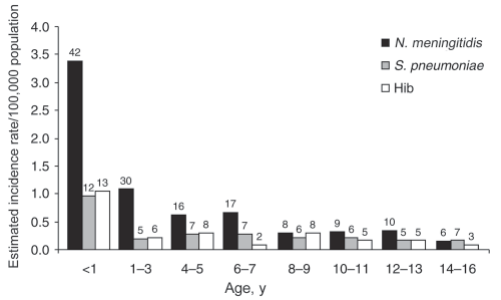
Distribution of bacteria causing childhood acute bacterial meningitis in different age groups. *Neisseria meningitidis* was the most common cause of meningitis, and the highest estimated incidence was in children <1 year of age for all 3 bacteria. The number of cases is indicated above each bar. *S. pneumoniae*, *Streptococcus pneumoniae*; Hib, *Haemophilus influenzae* type b.

Of the 408 cases, bacterial meningitis was confirmed by PCR, culture, or latex agglutination test in 243 (59.6%) patients. Regional incidence rates of laboratory-confirmed meningitis were estimated as ranging from 1/100,000 population in the Black Sea region to 10.9/100,000 population in the Southeast Anatolia region (Figure 1). Nationwide, the highest incidence was in children 1–12 months of age and was slightly more common in boys. The boy-to-girl ratio of the confirmed cases was 1.3:1, and the age distribution was as shown in Figure 2.

Overall, the diagnosis of acute bacterial meningitis was confirmed with CSF culture in 41 (17%) of 243 cases, with latex agglutination test in 56 (23%), and with PCR in 243 (100%) (Table). Latex agglutination test was positive in 37 cases for *N. meningitidis*, in 10 cases for Hib, and in 9 cases for *S. pneumoniae*.

**Table:** Laboratory confirmation of bacterial meningitis*

Category	PCR positive (n = 243)	PCR negative (n = 165)	Total (n = 408)
CSF culture			
Positive	41	0	41
Negative	202	165	367
Latex agglutination test			
Positive	53	3	56
Negative	190	162	352

*Specimens positive for individual assays and combinations of assays. CSF, cerebrospinal fluid.

Where data were available, 7 (17%) of 41 cases with positive CSF culture and 111 (54.9%) of 202 cases with negative CSF culture had a history of use of antimicrobial agent(s) before lumbar puncture, which may account for the relatively low diagnosis rate by using this technique. *N. meningitidis* was reported in 23 cases, *S. pneumoniae* was reported in 12, and Hib was reported in 6 cases as positive in CSF culture.

Blood culture was positive in 12 (4.9%) of 243 cases—4 each of *N. meningitidis*, Hib, and *S. pneumoniae*. Phenotyping of 21 available isolates indicated W135:2a:P1.5,2 (5 cases), A:21:NT:P1.10 (1 case), B:NT:P1.12,4 (2 cases), B:22:NT:NT (2 cases), B:NT:NT:P1.14 (2 cases), B:15:P1.7,16 (3 cases), B:14:NT:P1.13 (2 cases), B:15:NT:P1.16 (2 cases), X:NT:P1.7,1 (1 case), Y: NT:P1.5:NT (1 case).

**Figure 3:**
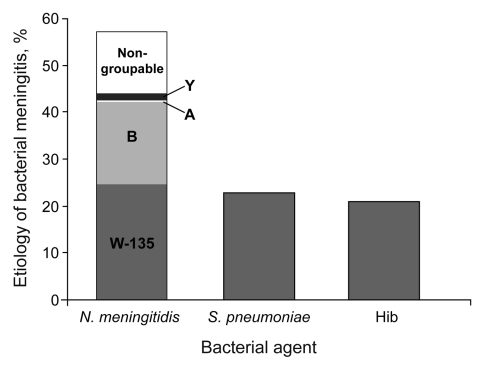
Distribution of etiology of acute bacterial meningitis in Turkey detected by using PCR analysis. Of 243 PCR-confirmed cases, 138 (56.5%) were attributable to *Neisseria meningitidis*, 55 (22.5%) to *Streptococcus pneumoniae*, and 50 (20.5%) to *Haemophilus influenzae* type b (Hib).

PCR analysis was by far the most reliable method of confirming bacterial meningitis, accounting for all confirmed cases with 243 positive results. In these PCR-positive samples, 138 (56.5%) were attributable to *N. meningitidis*, 55 (22.5%) to *S. pneumoniae*, and 50 (20.5%) to Hib (Figure 3). Of the 408 patients, 118 (48.5%) of 243 cases with positive PCR and 96 (58.2%) of 165 cases with negative PCR had received antibacterial drugs in the week before CSF sampling.

In the evaluation of the bacterial agents among the 7 different geographic regions of the country, *N. meningitidis* was the most common cause of acute bacterial meningitis in all regions except the Mediterranean region, located on the southern coast of Turkey. Here *S. pneumoniae* were the prominent bacteria, and *N. meningitidis* were detected in only 2 cases.

Comparison of the incidence of *N. meningitidis*, *S. pneumoniae*, and Hib among different age groups demonstrated that *N. meningitidis* was the prominent bacterial agent causing acute bacterial meningitis, especially in children <7 years of age (Figure 2). The highest incidence was detected during the first year of life for all 3 bacteria.

CSF findings were recorded for 368 (90.2%) of 408 CSF samples sent to the Central Laboratory. As a mean, in PCR-negative samples, CSF protein level was significantly lower (70.2 vs. 130.4 mg/dL; p = 0.003) and glucose level was significantly higher (55.6 vs. 41.3 mg/dL; p = 0.01) than in PCR-positive samples. Total cell counts were not significantly different in PCR-negative and -positive samples (157.9 and 211.1/mm^3^; p>0.05), but polymorphonuclear cell count was significantly higher in PCR-positive samples (8,284.3 vs. 38.5/mm^3^; p = 0.001). Among the PCR-positive samples, total cell and polymorphonuclear cell counts were not significantly different between samples positive for *N. meningitidis*, *S. pneumoniae*, and Hib (p>0.05).

### Meningococcal Epidemiology

Among the samples that were positive for *N. meningitidis* following PCR analysis, serogroup W-135 was the cause of most infections; 59 (42.7%) cases were serogroup W-135, 43 (31.1%) were serogroup B, 3 (2.2%) were serogroup Y, and 1 (0.7%) was serogroup A. There were no cases with a positive result for serogroup C (this was also the case following analysis of CSF culture-positive samples) and in 32 (23.2%) *N. meningitidis*–positive samples the serogroup could not be determined by the PCR assay.

**Figure 4:**
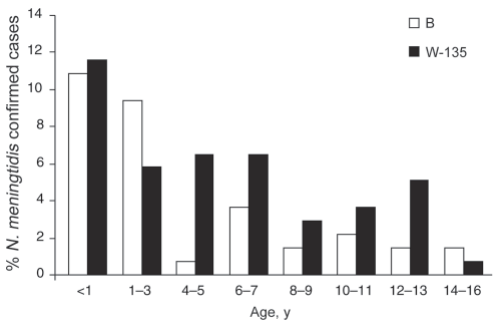
Distribution of predominant *Neisseria meningitidis* serogroups in different age groups. Serogroups W-135 and B caused 42.7% and 31.1% of all meningococcal infections, respectively. W-135 was the most common cause of meningococcal infection in all but 2 age groups analyzed.

Analysis by age reveals the greatest meningococcal disease incidence is in children <3 years of age, particularly infants <1 year of age. The numbers of cases caused by the 2 most common *N. meningitidis* serogroups (serogroups W-135 and B) were similar in the most vulnerable age groups (<3 years of age), but W-135 was more common in children 4–16 years of age (Figure 4).

**Figure 5:**
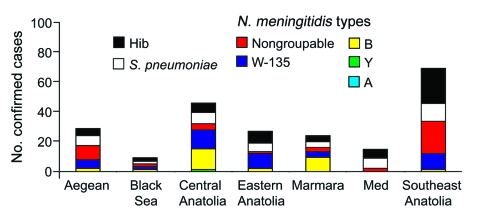
Etiology of confirmed cases of bacterial meningitis in different geographic regions. W-135 was the most prominent *Neisseria meningitidis* serogroup in the Southeast Anatolia, Aegean, Eastern Anatolia, and Black Sea regions. The percentages of cases caused by *Streptococcus pneumoniae* and *Haemophilus influenzae* type b (Hib) are also shown.

Etiologic and meningococcal serogroup distribution among the different geographic regions is illustrated in Figure 5. *N. meningitidis* serogroup W-135 was more prominent than the other meningococcal serogroups in the Southeast Anatolia, Aegean (Western Turkey), Eastern Anatolia, and Black Sea regions. *N. meningitidis* serogroup B was much more common in the Marmara region (northwestern Turkey), and in the Central region; the numbers of serogroup B and serogroup W-135 cases were similar. The Mediterranean region had 2 *N. meningitidis*–positive samples; both were nongroupable by PCR analysis.

## Discussion

In this study, 243 cases of laboratory-confirmed acute bacterial meningitis were recorded. Because our study centers provide service to 32% of the population of Turkey, extrapolation from the number of cases recorded suggests that 759 acute bacterial meningitis cases (excluding neonatal cases) per year occur in the whole country. The population of children 1 month through 16 years of age was calculated as 21.6 million. Therefore, the annual incidence of acute bacterial meningitis was estimated as 3.5 cases/100,000/year. Although similar to incidence rates reported from other countries without routine vaccination against *N. meningitidis, S. pneumoniae*, and Hib (*3*,*4*,*10*,*30*), this value likely represents a lower limit estimate of the true disease incidence, given the inherent limitations of hospital-based surveillance. Furthermore, the specific role of these 3 most common bacterial causes of acute bacterial meningitis varies between regions.

An accurate laboratory confirmation of the etiology in acute bacterial meningitis is essential to provide optimal patient therapy, appropriate case contact management, and reasoned public health actions. Prospectively, it also provides information upon which to base decisions regarding immunization programs, especially for countries without routine vaccination against the main acute bacterial meningitis pathogens (*28*,*31*). Although bacterial culture is considered to be the standard method, the negative effect of prior antimicrobial drug use on its sensitivity necessitates nonculture techniques for diagnosis. Among nonculture diagnostic tests, PCR is the most accurate and reliable method, especially among patients with a history of antimicrobial drug use before spinal tap (*32*). This finding was evident in the present study, in which PCR analysis was the most sensitive method, confirming 243 cases (59.6%) among 408 children meeting the case definition for bacterial meningitis (100% of all cases that were confirmed by any method). Using other methods that are more sensitive may increase the rate of laboratory confirmation.

Several reports review the rates of bacterial causes of acute bacterial meningitis from many different countries, based on CSF cultures. Some factors, such as previous antimicrobial drug treatment, interfere with the recovery of microorganisms from CSF. In our study, bacterial isolation was only possible in 41(16.8%) of 243 of confirmed cases. However, the most important factor for this low positive ratio in culture was likely prior antimicrobial drug use, as 118 case-patients received such treatment before the lumbar puncture (7/41; 17% in culture-positive case-patients and 111 (54.9%) of 202 in culture-negative case-patients). In patients with acute bacterial meningitis, blood cultures can be used in the etiologic diagnosis in up to 80% of cases since the bacteria generally invade meningeal membranes following bacteremia (*3*,*10*,*33*). In our study, however, only 12 (4.9%) of 243 case-patients who had blood culture tests returned positive results. This finding may also be related to the previous antimicrobial drug use.

Previous reports suggested that *S. pneumoniae* and *N. meningitidis* serogroups A and B would be the most common bacteria causing acute bacterial meningitis in Turkey (*25*,*34*–*36*). Serogroup W-135 meningococcus was isolated for the first time in Turkey in an asymptomatic healthy preschool child in 2001 (*23*), and the first patient with meningitis caused by serogroup W-135 was reported in 2003 (*24*). In this study *N. meningitidis,* especially serogroup W-135, was responsible for most of the cases observed, with serogroup B the second most common. Only a small number of serogroup A or Y cases were noted, and no serogroup C cases were observed. These data are in contrast to those from many parts of Europe, where serogroups B and C dominate the epidemiologic landscape. In Turkey, meningococcal disease caused by serogroup W-135 has increased from 1 case in 2003 to 59 cases or 42.7% of all laboratory-confirmed *N. meningitidis* cases in children, in this study, during 2005/06. A dramatic increase in serogroup Y disease has been documented in the United States during the last decade, but this has been over a longer period.

Most of the Turkish population is Muslim, and ≈150,000 pilgrims travel annually to Saudi Arabia for the Hajj. Since 2002, all Turkish pilgrims have received a quadrivalent meningococcal polysaccharide vaccine before travel. Although this vaccine generates a robust immune response against serogroup W-135, in contrast to what has been demonstrated for serogroup C meningococcal conjugate vaccines (*37*), meningococcal polysaccharide vaccines are not thought to reliably prevent asymptomatic carriage. A study from the United States reported that 0.8% of returning vaccinated pilgrims in 2001 were carrying W-135, whereas no pilgrims carried this serogroup upon departure from the United States (*38*). Therefore, the rapid rise in the proportion of cases caused by serogroup W-135 may be attributable to transmission from pilgrims returning from the Hajj carrying this particular serogroup. Although not definitive, this conclusion is further supported by the finding that all serogroup W-135 isolates available for phenotypic characterization were identical to the Hajj-associated clone, W135:2a:P1.5,2 (*20*). Because strains with this serologic profile have not been uniquely associated with the Hajj outbreak (*39*), additional typing data (e.g., multilocus sequence typing or multilocus enzyme electrophoresis), and epidemiologic investigations will be required to support this hypothesis. Therefore, it remains speculative that the increased W-135 disease in Turkey may be caused by spread of Hajj epidemic strain through carriage and transmission by pilgrims.

This study demonstrates the need for good quality, continued surveillance of bacterial meningitis cases, as well as the etiology and epidemiology of the causative bacteria. Only by accurately monitoring meningococcal epidemiology will effective vaccination policies be developed. The bacterial meningitis epidemiologic landscape is not static, and the causative agents change with time and across regions of the world. This study has demonstrated that the relative contribution of serogroup W-135 to the meningococcal disease incidence in Turkey is increasing, which is in contrast to the rest of Europe. Turkey may remain isolated in terms of W-135 disease incidence or it may represent the beginning of epidemiologic change in Eastern Europe. This possibility should be investigated in greater depth and monitored prospectively. Introduction of vaccines can dramatically reduce the meningitis disease incidence, but these vaccines must be targeted against the correct bacteria and, where relevant, the correct bacterial serogroup. The choice of meningococcal conjugate vaccines in Turkey will need to include coverage for serogroup W-135; introduction of such a vaccine would be helpful in protecting the Turkish population from this invasive bacterial meningitis. Moreover, it may also be prudent to switch the meningococcal vaccine used for pilgrims to conjugated vaccine to prevent the carriage of the microorganism by pilgrims.

## References

[1] World Health Organization. World health report. 2000 [cited 2008 May 1]. Available from http://www.who.int/whr/2000/en/index.html

[2] Jodar L, Feavers IM, Salisbury D, Granoff DM. Development of vaccines against meningococcal disease. Lancet. 2002;359:1499–508. 10.1016/S0140-6736(02)08416-7 11988262

[3] Feigin RD, Pearlman E. Bacterial meningitis beyond the neonatal period. In: Feigin RD, Demler GJ, Cherry JD, Kaplan SL, editors. Textbook of pediatric infectious diseases. 5th ed. Philadelphia: Saunders; 2004. p. 443–74.

[4] Saez-Llorens X, McCracken GH Jr. Bacterial meningitis in children. Lancet. 2003;361:2139–48. 10.1016/S0140-6736(03)13693-8 12826449

[5] Baraff LJ, Lee SI, Schriger DL. Outcomes of bacterial meningitis in children: a meta-analysis. Pediatr Infect Dis J. 1993;12:389–94. 10.1097/00006454-199305000-00008 8327300

[6] Grandgirard D, Leib SL. Strategies to prevent neuronal damage in paediatric bacterial meningitis. Curr Opin Pediatr. 2006;18:112–8. 10.1097/01.mop.0000193292.09894.b7 16601488

[7] Peltola H. Worldwide *Haemophilus influenzae* type b disease at the beginning of the 21st century: global analysis of the disease burden 25 years after the use of the polysaccharide vaccine and a decade after the advent of conjugates. Clin Microbiol Rev. 2000;13:302–17. 10.1128/CMR.13.2.302-317.2000 10756001 PMC100154

[8] Centers for Disease Control and Prevention. Progress toward elimination of *Haemophilus influenzae* type b invasive disease among infants and children: United States, 1998–2000. MMWR Morb Mortal Wkly Rep. 2002;51:234–9. 11925021

[9] Melegaro A, Edmunds WJ, Pebody R, Miller E, George R. The current burden of pneumococcal disease in England and Wales. J Infect. 2006;52:37–48. 10.1016/j.jinf.2005.02.008 16368459

[10] Pathan N, Faust SN, Levin M. Pathophysiology of meningococcal meningitis and septicaemia. Arch Dis Child. 2003;88:601–7. 10.1136/adc.88.7.601 12818907 PMC1763171

[11] World Health Organization. Outbreak news. Meningococcal disease, African meningitis belt, epidemic season 2006. Wkly Epidemiol Rec. 2006;81:119–20. 16673512

[12] Anderson MS, Glode MP, Smith AL. Meningococcal disease. In: Feigin RD, Demler GJ, Cherry JD, Kaplan SL, editors. Textbook of pediatric infectious diseases. 5th ed. Philadelphia: Saunders; 2004. p. 1265–79.

[13] Jelfs J, Munro R. Epidemiology of meningococcal disease in Australia. J Paediatr Child Health. 2001;37:3–6. 10.1046/j.1440-1754.2001.00680.x 11885734

[14] Ramsay ME, Andrews N, Kaczmarski EB, Miller E. Efficacy of meningococcal serogroup C conjugate vaccine in teenagers and toddlers in England. Lancet. 2001;357:195–6. 10.1016/S0140-6736(00)03594-7 11213098

[15] Snape MD, Pollard AJ. Meningococcal polysaccharide-protein conjugate vaccines. Lancet Infect Dis. 2005;5:21–30. 10.1016/S1473-3099(04)01251-4 15620558

[16] Nicolas P, Ait M’barek N, Al-Awaidy S, Al BS, Sulaiman N, Issa M, et al. Pharyngeal carriage of serogroup W135 *Neisseria meningitidis* in Hajjees and their family contacts in Morocco, Oman and Sudan. APMIS. 2005;113:182–6. 10.1111/j.1600-0463.2005.apm1130305.x 15799761

[17] Lind I, Berthelsen L. Epidemiology of meningococcal disease in Denmark 1974–1999: contribution of the laboratory surveillance system. Epidemiol Infect. 2005;133:205–15. 10.1017/S0950268804003413 15816145 PMC2870239

[18] Gray SJ, Trotter CL, Ramsay ME, Guiver M, Fox AJ, Borrow R, Epidemiology of meningococcal disease in England and Wales 1993/94 to 2003/04: contribution and experiences of the Meningococcal Reference Unit. J Med Microbiol. 2006;55:887–96. 10.1099/jmm.0.46288-0 16772416

[19] Rosenstein NE, Perkins BA, Stephens DS, Lefkowitz L, Cartter ML, Danila R, The changing epidemiology of meningococcal disease in the United States, 1992–1996. J Infect Dis. 1999;180:1894–901. 10.1086/315158 10558946

[20] Taha MK, Achtman M, Alonso JM, Greenwood B, Ramsay M, Fox A, Serogroup W135 meningococcal disease in Hajj pilgrims. Lancet. 2000;356:2159. 10.1016/S0140-6736(00)03502-9 11191548

[21] Novelli VM, Lewis RG, Dawood ST. Epidemic group A meningococcal disease in Haj pilgrims [letter]. Lancet. 1987;330:863. 10.1016/S0140-6736(87)91056-7 2889067

[22] Al-Mazrou YY, Al-Jeffri MH, Abdalla MN, Elgizouli SA, Mishskas AA. Changes in epidemiological pattern of meningococcal disease in Saudi Arabia. Does it constitute a new challenge for prevention and control? Saudi Med J. 2004;25:1410–3. 15494812

[23] Bakir M, Yagci A, Ulger N, Akbenlioglu C, Ilki A, Soyletir G. Asymtomatic carriage of *Neisseria meningitidis* and *Neisseria lactamica* in relation to *Streptococcus pneumoniae* and *Haemophilus influenzae* colonization in healthy children: apropos of 1400 children sampled. Eur J Epidemiol. 2001;17:1015–8. 10.1023/A:1020021109462 12380714

[24] Doganci L, Baysallar M, Saracli MA, Hascelik G, Pahsa A. *Neisseria meningitidis* W135, Turkey. Emerg Infect Dis. 2004;10:936–7. 15200836 10.3201/eid1005.030572 PMC3323204

[25] Kilic A, Urwin R, Li H, Saracli MA, Stratton CW, Tang YW. Clonal spread of serogroup W135 meningococcal disease in Turkey. J Clin Microbiol. 2006;44:222–4. 10.1128/JCM.44.1.222-224.2006 16390974 PMC1351935

[26] Berkley JA, Versteeg AC, Mwangi I, Lowe BS, Newton CR. Indicators of acute bacterial meningitis in children at a rural Kenyan district hospital. Pediatrics. 2004;114:e713–9. 10.1542/peds.2004-0007 15574603

[27] Abdillahi H, Poolman JT. Typing of group-B *Neisseria meningitidis* with monoclonal antibodies in the whole-cell ELISA. J Med Microbiol. 1988;26:177–80. 3134550

[28] Taha MK. Simultaneous approach for nonculture PCR-based identification and serogroup prediction of *Neisseria meningitidis.* J Clin Microbiol. 2000;38:855–7. 10655397 10.1128/jcm.38.2.855-857.2000 PMC86222

[29] Tsolia MN, Theodoridou M, Tzanakaki G, Kalabalikis P, Urani E, Mostrou G, The evolving epidemiology of invasive meningococcal disease: a two-year prospective, population-based study in children in the area of Athens. FEMS Immunol Med Microbiol. 2003;36:87–94. 10.1016/S0928-8244(03)00083-X 12727371

[30] Saravolatz LD, Manzor O, VanderVelde N, Pawlak J, Belian B. Broad-range bacterial polymerase chain reaction for early detection of bacterial meningitis. Clin Infect Dis. 2003;36:40–5. 10.1086/345438 12491200

[31] Gray LD, Fedorko DP. Laboratory diagnosis of bacterial meningitis. Clin Microbiol Rev. 1992;5:130–45. 1576585 10.1128/cmr.5.2.130 PMC358232

[32] Tzanakaki G, Tsopanomichalou M, Kesanopoulos K, Matzourani R, Sioumala M, Tabaki A, Simultaneous single-tube PCR assay for the detection of *Neisseria meningitidis, Haemophilus influenzae* type b and *Streptococcus pneumoniae.* Clin Microbiol Infect. 2005;11:386–90. 10.1111/j.1469-0691.2005.01109.x 15819865

[33] Luby JP. Infections of the central nervous system. Am J Med Sci. 1992;304:379–91. 10.1097/00000441-199212000-00010 1456278

[34] Berkman E, Ozben G. Meningococcic meningitis epidemic in Ankara [in Turkish]. Mikrobiyol Bul. 1982;16:101–6. 6815435

[35] Coskun S, Yanikyurek S, Agzitemiz M. Incidence of epidemiological meningitis in Aegean region. Turk J Infect. 1990;4:431–5.

[36] Gazi H, Surucuoglu S, Ozbakkaloglu B, Akcali S, Ozkutuk N, Degerli K, Oropharyngeal carriage and penicillin resistance of *Neisseria meningitidis* in primary school children in Manisa, Turkey. Ann Acad Med Singapore. 2004;33:758–62. 15608834

[37] Maiden MC, Stuart JM; UK Meningococcal Carriage Group. Carriage of serogroup C meningococci 1 year after meningococcal C conjugate polysaccharide vaccination. Lancet. 2002;359:1829–31. 10.1016/S0140-6736(02)08679-8 12044380

[38] Centers for Disease Control and Prevention. Assessment of risk for meningococcal disease associated with Hajj 2001. MMWR Morb Mortal Wkly Rep. 2001;50:221–2. 11300626

[39] Mayer LW, Reeves MW, Al-Hamdan N, Sacchi CT, Taha MK, Ajello GW, Outbreak of W135 meningococcal disease in 2000: not emergence of a new W135 strain but clonal expansion within the electrophoretic type-37 complex. J Infect Dis. 2002;185:1596–605. 10.1086/340414 12023765

